# Multimodal Guided Self-Help Exercise Program to Prevent Speech, Swallowing, and Shoulder Problems Among Head and Neck Cancer Patients: A Feasibility Study

**DOI:** 10.2196/jmir.2990

**Published:** 2014-03-06

**Authors:** Ingrid C Cnossen, Cornelia F van Uden-Kraan, Rico NPM Rinkel, IJke J Aalders, Cees JT de Goede, Remco de Bree, Patricia Doornaert, Derek HF Rietveld, Johannes A Langendijk, Birgit I Witte, C Rene Leemans, Irma M Verdonck-de Leeuw

**Affiliations:** ^1^VU University Medical CenterDepartment of Otolaryngology - Head and Neck SurgeryVU University Medical CenterAmsterdamNetherlands; ^2^VU UniversityDepartment of Clinical PsychologyVU UniversityAmsterdamNetherlands; ^3^VU University Medical CenterDepartment of PhysiotherapyVU University Medical CenterAmsterdamNetherlands; ^4^VU University Medical CenterDepartment of Radiation OncologyVU University Medical CenterAmsterdamNetherlands; ^5^University Medical Center, University of GroningenDepartment of Radiation OncologyUniversity Medical Center Groningen, University of GroningenGroningenNetherlands; ^6^VU University Medical CenterDepartment of Epidemiology and BiostatisticsVU University Medical CenterAmsterdamNetherlands

**Keywords:** eHealth, self-care, head and neck cancer, exercise, speech, swallowing, shoulder, surgery, radiotherapy, chemotherapy

## Abstract

**Background:**

During a 6-week course of (chemo)radiation many head and neck cancer patients have to endure radiotherapy-induced toxicity, negatively affecting patients’ quality of life. Pretreatment counseling combined with self-help exercises could be provided to inform patients and possibly prevent them from having speech, swallowing, and shoulder problems during and after treatment.

**Objective:**

Our goal was to investigate the feasibility of a multimodal guided self-help exercise program entitled Head Matters during (chemo)radiation in head and neck cancer patients.

**Methods:**

Head and neck cancer patients treated with primary (chemo)radiation or after surgery were asked to perform Head Matters at home. This prophylactic exercise program, offered in three different formats, aims to reduce the risk of developing speech, swallowing, shoulder problems, and a stiff neck. Weekly coaching was provided by a speech and swallowing therapist. Patients filled out a diary to keep track of their exercise activity. To gain insight into possible barriers and facilitators to exercise adherence, reports of weekly coaching sessions were analyzed by 2 coders independently.

**Results:**

Of 41 eligible patients, 34 patients were willing to participate (83% uptake). Of participating patients, 21 patients completed the program (64% adherence rate). The majority of participants (58%) had a moderate to high level of exercise performance. Exercise performance level was not significantly associated with age (*P*=.50), gender (*P*=.42), tumor subsite (*P*=1.00) or tumor stage (*P*=.20), treatment modality (*P*=.72), or Head Matters format (Web-based or paper) (*P*=1.00). Based on patients’ diaries and weekly coaching sessions, patients’ perceived barriers to exercise were a decreased physical condition, treatment-related barriers, emotional problems, lack of motivation, social barriers, and technical problems. Patients’ perceived facilitators included an increased physical condition, feeling motivated, and social and technical facilitators.

**Conclusions:**

Head Matters, a multimodal guided self-help exercise program is feasible for head and neck cancer patients undergoing (chemo)radiation. Several barriers (decreased physical condition, treatment-related barriers) and facilitators (increased physical condition, feeling motivated) were identified providing directions for future studies. The next step is conducting a study investigating the (cost-)effectiveness of Head Matters on speech, swallowing, shoulder function, and quality of life.

## Introduction

Head and neck cancers (HNC) in the oral cavity, nasopharynx, oropharynx, hypopharynx, and the larynx represent 5% of all cancers. About 2800 new cases are reported in the Netherlands each year. Treatment intensification using multimodality approaches, such as accelerated radiotherapy (RT), concomitant chemotherapy, and surgery with adjuvant radiotherapy with or without chemotherapy result in a significant improvement in loco-regional control and overall survival [1-3]. During a 6-week course of RT, many patients have to endure radiotherapy-induced toxicity such as oral mucositis, pain, salivary changes, dry mouth, skin toxicity, hoarseness, swallowing problems, trismus, fibrosis in the orofacial region, throat, neck and shoulders, and stiffness and pain in the neck and shoulders [4-15]. These acute side effects of radiation result in a significant symptom burden and interfere with normal physiologic functions and daily activities, such as chewing, swallowing and speech, and related social withdrawal and psychological distress, negatively affecting patients’ quality of life [16,17]. Swallowing problems are among the most cited functional impairments after chemoradiotherapy [18-20] with an estimated prevalence of 43% to 64% [21,22]. These results emphasize the importance of prevention, monitoring, and management of swallowing dysfunction as an integral part of treatment protocols [23].

It is expected that fewer speech and swallowing problems persist when these acute side effects of radiation are prevented and/or managed in an early stage [24,25]. Pretreatment counseling by a speech and swallowing therapist (ST) could be provided to inform the patient and family on possible speech and swallowing problems that may occur during and after treatment [26]. Patients should, for example, be informed about the importance of continuing to swallow throughout their courses of (chemo)radiation ([C]RT), because inactivity of the swallowing muscles may lead to disuse atrophy, and then lead to future, temporary inability to consume food orally and long-term feeding tube dependency [19,27,28]. In order to possibly prevent atrophy of the head and neck muscles, to maintain speech and swallowing function, and to improve functional swallowing outcome and swallowing-specific quality of life following (C)RT, counseling combined with exercise prescription should be provided prior to (C)RT [29-34].

Research is, however, still in an early stage and much is unknown [35,36]. As a result, not all patients with HNC undergoing (C)RT are prescribed a standardized exercise program as a preventive measure [37]. Given the burdensome period of (C)RT for HNC patients, there is an urgent need for an easily accessible prophylactic education and exercise program, countering the radiation fibrosis, and safeguarding patients against additional consults with health care professionals during (C)RT. A multimodal self-help program is expected to enhance reach by overcoming logistical and financial barriers both on the part of health care providers and patients [38,39]. To our knowledge, there are no self-help programs offered with remote coaching, targeting prevention of deterioration of speech, swallowing, and shoulder function.

Therefore we developed Head Matters (HM), a multimodal guided self-help exercise program for HNC patients during (C)RT. The aims of the present feasibility study were (1) to explore uptake, adherence, and exercise performance (by exercise levels and exercise categories) of the guided self-help exercise program HM in HNC patients, (2) to explore predictors of exercise performance, and (3) to gain insight into barriers and facilitators to exercise adherence.

## Methods

### Description of the Self-Help Exercise Program Head Matters (HM)

HM was developed by a team of health care professionals consisting of speech and swallowing therapists, otolaryngologists, head and neck surgeons, radiation oncologists, and a physiotherapist. HM comprises one face-to-face pretreatment counseling session, on the first day of (C)RT, to inform the patient of possible speech, swallowing, and shoulder problems during treatment and to encourage patients to maintain speech, swallowing, and shoulder function during treatment. HM consists of a 15-minute per day program with four categories of prophylactic exercises: (1) exercises to maintain mobility of the head, neck, and shoulders, (2) exercises to optimize and maintain swallowing function, (3) exercises to optimize and maintain vocal health and vocal function, and (4) exercises to optimize and maintain speech function and functional communication. Coaching is offered in weekly 10-minute coaching sessions by an experienced ST by phone or email.

**Figure 1:**
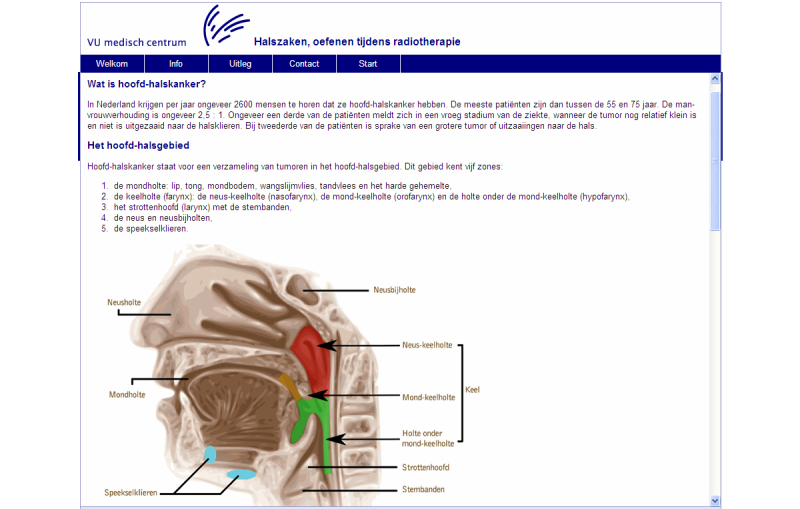
Screenshot of Head Matters: general information about head and neck cancer.

**Figure 2:**
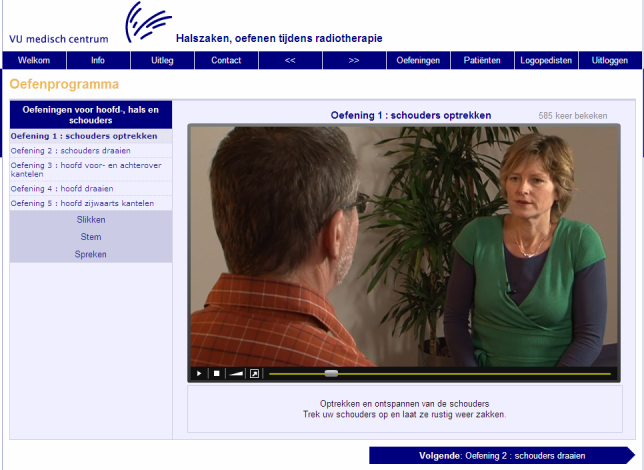
Screenshot of Head Matters. Exercise 1: Move your shoulders up and down.

Because the target group (HNC patients) does not have equal access to the Internet, HM is available in three different formats. Both the online format and booklet format offer general information about HNC and its treatment, with written descriptions of the exercises, and with photo and video examples of the exercises either offered online [40] or by means of a 15-minute instructional DVD. The third format consists of a 2-paged A4 leaflet that offers only a written description of the exercises. Multimedia Appendix 1 shows an overview of exercise categories and the three formats of HM. Examples of screenshots of the online format are shown in Figures 1 and 2.

### Study Sample and Procedure

HNC patients treated at the VU University Medical Center Amsterdam, the Netherlands, had to fulfil the following criteria to be included in this feasibility study: (1) age ≥18 years, (2) HNC originating in the oral cavity, oropharynx, hypopharynx, or larynx, (3) stage I-IV cancer, according to the Union for International Cancer Control (UICC) TNM-classification system, (4) no distant metastases, (5) radiation, (C)RT or postoperative (C)RT, and (6) absence of any psychological, familial, sociological, or geographical condition potentially hampering compliance with the study protocol. Three radiation oncologists introduced the study to eligible patients who met the inclusion criteria based on medical chart reviews, and based on the conversation with the patient during a regular consultation. If a patient expressed interest in participation, he or she was approached by the researcher for further details about the study. The patient’s written informed consent form was obtained.

Patients were invited to perform HM at home, at least once a day. If a patient was willing to perform the exercises, a 15-minute face-to-face instruction session by an ST was planned on the first day of (C)RT. Safety was ensured by demonstrating each exercise appropriately, by giving the participants adequate instructions on each exercise, and by providing an instruction leaflet, an instruction booklet (with DVD), or a log-in code to activate an account for the website [40]. Postoperative participants were offered the HM program on a leaflet. Patients with Internet access and treated with (C)RT were offered the HM online format and the HM instruction booklet with general information about HNC, written instructions, and photo demonstration. Patients without Internet access and treated with (C)RT were offered the HM instruction booklet with general information about HNC, written instructions, and photo and video demonstration (on DVD).

Patients were asked to fill out a diary on paper or online for 6 weeks. In their diaries, patients noted which exercises they performed (of the four categories), and the frequency of exercising (1, 2, or 3 times per day.)

During 6 weeks of exercising with (C)RT, subjects participated in weekly 10-minute coaching sessions by an ST by phone or email to maintain motivation and to help them to achieve adherence to the HM protocol. During these coaching sessions, open-ended questions about general well-being were asked (“How are you?”) and questions about exercise performance (“Could you tell me something about your exercise frequency this week?”, and “Could you name any reasons (not) to exercise?”). The ST took a supportive role, while actively asking the participants for further explanations of their answers when necessary. During these weekly coaching sessions, notes were taken.

The study was conducted according to regular procedures of the local ethical committee of the VU University Medical Center, Amsterdam.

### Measures

Demographic (ie, gender, age) and clinical (ie, tumor subsite and stage, treatment modality) information of participating patients was extracted from the hospital information system.

### Uptake

Uptake of HM addressed how many patients were willing to start HM during (C)RT (uptake percentage).

### Adherence

Adherence concerned the degree to which HNC patients followed HM at least once a day during 6 weeks of (C)RT and was assessed in two ways: (1) patient-completed diaries, and (2) percentage of patients who started and kept up exercising for 6 weeks.

### Exercise Performance Level

Patient-completed diaries were used to identify exercise performance levels. A low level of exercise performance consisted of an exercise performance of all exercise categories during 6 weeks at most once a day on average (range 0-168). A moderate level consisted of an exercise performance of all categories during 6 weeks between once and twice a day on average (range 169-336). A high level of exercise performance was defined as an exercise performance of all exercise categories during 6 weeks at least twice a day on average (range 337-504).

To gain insight into which exercises were performed most often, the diaries were analyzed in more detail regarding the frequency of exercising (1-3 times) and type of exercise (four categories). Exercise performance by exercise format was defined as how well the prescribed exercise regimen was followed by patients, following a specific format (online exercising or exercising by leaflet or booklet).

### Feasibility

HM is defined to be feasible in case of an uptake percentage >50%, adherence rate >50%, and when >50% of the patients perform at least the minimum number of exercises (168) during 6 weeks (moderate or high performance level). This definition of feasibility is based on adherence rates reported in previous research [32,37]. In a retrospective study, adherence to unsupervised, home-based swallowing exercises is quite low, ranging from 13% for full adherence to 32% for partial adherence [37]. Van der Molen [32] retrospectively assessed adherence via two self-report items estimating duration of adherence in days and familiarity with exercises 10 weeks after treatment. They found that 14% of the total sample reported doing exercises every day during the entire radiation treatment and follow-up period and that 57% stopped their exercises after an average of 3.5 weeks.

### Barriers and Facilitators to Exercise

Reports of the coaching sessions were used to identify patients’ perceived barriers and facilitators to perform HM during (C)RT.

### Data Analysis

Quantative data were analyzed using IBM SPSS Statistics for Windows, version 20. Descriptive statistics were used to summarize the sociodemographics and clinical characteristics of the study participants and the data on uptake, adherence, and performance level of HM.

Patients were categorized regarding exercise performance level (low, moderate, high), and age (≤60 years vs ≥61 years, based on median split), tumor subsite (oral cavity, oropharynx, hypopharynx, larynx, other), tumor stage (I, II, III, IV), treatment (RT, chemoradiation [CRT], surgery, and [C]RT), and format of HM (leaflet, booklet, online). Fisher’s Exact tests were used to determine differences in exercise performance level (performance level low vs moderate/high) regarding age, gender, tumor subsite (oral cavity/oropharynx vs hypopharynx/larynx), tumor stage (stage I/II vs III/IV), treatment modality ([C]RT vs postoperative [C]RT), and format of HM (online vs leaflet vs booklet). For all analyses, *P*≤.05 was considered statistically significant.

Reports of the weekly coaching sessions were analyzed by 2 independent observers (IC and CvU). Both coders separately read all reports of the weekly coaching sessions several times to familiarize themselves with the data. Barriers and facilitators for exercising were selected and coded independently into categories. Subsequently, the coders met to discuss their findings and resolve differences with the aim of reaching consensus, after which categories were refined, and subcategories were identified. The coders met regularly with a third coder (IV) to resolve disagreements in coding.

## Results

### Uptake and Participants’ Characteristics

In total, 41 eligible patients were referred to the study. Due to shortage of time, 7 of the 41 patients refused to participate; 34 patients agreed to participate and were enrolled in the study (83% uptake). One patient agreed to participate but died 1 week after giving written informed consent. Eleven postoperative patients (33%) received HM on a 2-paged leaflet, 11 patients (33%) chose to receive HM in a 28-page booklet format with photos and video examples on a DVD, and another group of 11 patients (33%) chose to receive HM online.

The mean age of the participants was 60 years (range 21-77). Of the 33 patients (76% male, and 24% female), one third of the patients was treated with RT (33%), one third of the patients was treated with CRT (33%), and one third with surgery (33%). After surgery, 7 patients received postoperative RT, and 4 patients received postoperative CRT (Multimedia Appendix 2).

In the planned face-to-face instruction session, 26 of the 33 patients (79%) received exercise instructions on day one of (C)RT. The other 7 patients (21%) received their exercise instructions 3-11 days earlier and started exercising before (C)RT started. In total, 33 patients filled out a diary.

### Adherence and Exercise Performance Level

Of the 33 patients who were interested in performing exercises, 21 patients started and kept up exercising for 6 weeks (64% adherence rate). Of the 33 patients, 14 patients (42%) were performing the exercises at a low level (exercise frequency range of 4-167 during 6 weeks), 10 patients (30%) were exercising at a moderate level (exercise frequency range of 196-332), and 9 patients (27%) were exercising at a high level (exercise frequency range of 372-495) (Multimedia Appendix 3).

### Exercise Performance by Exercise Category

**Figure 3:**
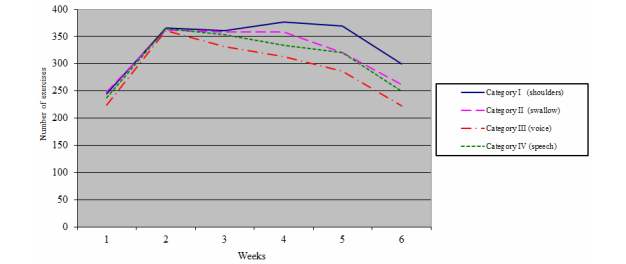
Weekly exercise performance by exercise category.

Figure 3 presents an overview of all 33 patients regarding the course of exercise through the 6 weeks of (C)RT. Patients most often performed the exercises to maintain mobility of the head, neck, and shoulders. The exercises least performed by patients were to optimize vocal health and maintain vocal function. During the first 2 weeks, exercise performance of all 33 patients increased from 224 to 366 exercises, on average. After the second week, a decline in exercise performance of all participants in all exercise categories was observed. During the sixth week of (C)RT, patients were still performing HM exercises, with an average of 259 times.

Based on these results, HM appears to be feasible in general, with an uptake percentage >50% (in the present study 83%), with an adherence rate >50% (in the present study 64%), and with a moderate to high performance level >50% of the patients performing exercises in all categories at least once a day on average (in the present study, 58% of the participants).

### Predictors of Exercise Performance

Exercise performance level was not significantly related to age (*P=*.50), gender (*P=*.42), tumor subsite (*P=*1.00), tumor stage (*P=*.20), or treatment modality (*P=*.72). Exercise performance level was also not significantly different regarding HM format (*P=*1.00).

###  Barriers to Exercise

#### Overview

From the analysis of reports of weekly coaching sessions, several barriers to perform HM emerged: a decreased physical condition, treatment-related barriers, emotional problems, lack of motivation, social barriers, and technical problems (Multimedia Appendix 4).

#### Decreased Physical Condition

During coaching sessions, participants commented that they did not perform the exercises because of oral complications and throat problems (eg, swallowing, speech and voice problems, limited mouth opening, skin and oral wounds, oral infections, saliva problems, swelling, taste problems, having a poor appetite, and dental extractions), as well as stiffness in the neck and shoulders. In addition, participants mentioned more general physical symptoms resulting from cancer or cancer treatment, such as pain, nausea, weight loss, and fatigue, which prevented them from performing the exercises.

#### Treatment-Related Barriers

Some participants indicated that daily travelling to the outpatient clinic for (C)RT or just the (C)RT itself was too time-consuming to perform exercises. Others mentioned feeling embarrassed having to perform (voice) exercises in a hospital ward during hospitalization for chemotherapy.

#### Emotional Problems

Some participants noted that they found it difficult to focus on and pay attention to HM due to emotional problems (eg, anxiety, worrying, having panic attacks, feeling scared).

#### Lack of Motivation

Some participants indicated that they did not feel motivated to exercise because of not experiencing any complaints. Others mentioned that they were not convinced that the exercises would help. Some did not feel motivated to perform the exercises at home and preferred face-to-face contact with an ST. Others commented that the exercise program would distract them from their daily routine or reported a lack of motivation because of a “perceived information overload” during treatment.

#### Social Barriers

Some participants reported problems combining HM at home and work situations. Especially informal caregivers and participants with job responsibilities could not find the time to exercise and felt not able to concentrate on the exercise program.

#### Technical Problems

With regard to technical issues, patients reported installation problems and were not able to see the demonstration videos on the computer. One participant indicated that the exercise repetitions on DVD took too much time, leading to boredom. Four participants mentioned that they lost their log-in password or forgot the website address and therefore could not see the exercise demonstrations on video.

### Facilitators to Exercise

#### Overview

Besides barriers, facilitators to perform HM during (C)RT emerged: an increased physical condition, a general sense of psychological well-being, feeling motivated, and social and technical facilitators (Multimedia Appendix 4).

#### Increased Physical Condition

Some participants mentioned that a regained vocal function, an improved appetite, and a decreased size of their tumor enabled them to perform HM. Others mentioned that an increased general physical condition (eg, regained energy) facilitated exercise performance.

#### General Sense of Psychological Well-Being

Some participants stated that a general sense of psychological well-being, expressed as feeling good and being good-humored, encouraged them to perform the exercises.

#### Feeling Motivated

Participants reported enhanced motivation to perform the exercises because the exercises were simple and easy to follow. A motivational facilitator for some of the participants was that they knew the exercises by heart and could therefore perform the exercises while taking a shower or while on their way to the hospital (in a taxi). Some reported that they enjoyed the exercises because they experienced them as relaxing. They indicated liking the swallowing strategies at breakfast, lunch, or dinner. Others stated feeling motivated because, by adhering to the exercise program, they felt able to contribute to their own recovery process. Some stated that they adapted the exercises to their own ability and decided to perform the exercises more carefully and slower than demonstrated, and in shorter sessions throughout the day.

Participants stated that they enjoyed the design of HM. They especially mentioned the face-to-face introduction of the exercises and weekly coaching sessions as motivational.

#### Social Facilitators

Participants indicated social support in the home situation to be an important facilitator. Some felt encouraged to exercise because they performed the exercises together with their partner and/or family. Others felt motivated because their partner and/or family reported improvement due to exercising, such as a better speech function. One participant reported performing (more of) the exercises while being off duty, while another performed (more of) the exercises during working hours.

#### Technical Facilitators

Online or DVD exercise demonstrations were indicated by participants as an enabler to perform the exercises (in the right way and at the same place) as instructed.

## Discussion

### Principal Findings

Results from this feasibility study indicated that the guided self-help exercise program HM is feasible among HNC patients undergoing primary or postoperative (C)RT with high uptake and reasonable adherence rates. The majority of the included patients performed at least the minimum number of exercises during 6 weeks (moderate or high performance level).

The majority of HNC patients in our study (34/41, 83%) responded positively to the offer of pretreatment counseling on exercises to maintain speech, swallowing, and shoulder function while undergoing (C)RT. While the efficacy of our guided self-help exercise program is yet to be demonstrated, the high uptake of HM suggests that this program may have addressed specific needs among the target population.

To understand the true benefits of an exercise program, the adherence rates of patients involved in such programs is one of the key issues [31]. In the present study, adherence rate to the home-based HM program was 64%. Adherence to similar prophylactic exercise programs targeting HNC patients varied between 14% (a home-based program) [32] and 68% (twice a week supervised hospital-based exercises, combined with home-based exercises) [32-34]. Results are, however, not fully comparable because HM, unlike the other interventions, is a self-help program. Self-help programs, especially those administered online, often suffer from non-adherence [41,42]. The adequate adherence rate found in our study suggests that an exercise program such as HM can be offered in a home-based self-help format, with the use of self-regulating strategies, including diary keeping, possibly enhancing motivation, and adherence.

We explored predictors of exercise performance in HNC patients willing to use a guided self-help exercise program during (C)RT with minimal therapist guidance, offered in three exercise formats. Initially, we developed a leaflet format, followed in a later stage by an online and booklet format of HM, including photo and video demonstration of the exercises. Although we expected the later formats would possibly lead to a higher exercise performance level, in the present study no relation between exercise performance level and exercise format (exercising online or exercising by leaflet or booklet) were found. The small sample size of this feasibility study, lack of randomization, and lack of statistical power limited the comparability of findings and may explain why exercise performance levels and exercise format were not related significantly. Furthermore, exercise performance levels were based on patient-completed diaries and may not truly reflect the user’s experience and dose. In the upcoming study on (cost-)effectiveness of HM, we will maintain the online and booklet formats. The use of Web-based diaries enables health care providers to send reminders to participants and may provide interactive Internet feedback tailored to each patient, improving adherence. Despite the high prevalence of Internet access in the Netherlands and advantages of eHealth interventions, including multimedia presentation, easy updating of the information provided and tailoring, we think a booklet format is still required [43]. Within a specific part of the population of HNC patients (with higher age and lower socioeconomic status) the percentage of patients for whom an online intervention is not eligible is deemed high, because of low eHealth literacy skills, concerns about Internet privacy, and/or preferences for using a booklet [44,45]. Furthermore, online exercise videos and exercise demonstrations on DVD help HNC patients to safely and properly perform the exercises. Although the adherence rate in the present study was adequate (64%), efforts to increase adherence rate are needed. Low or non-adherence has been proposed as a risk and a reason for a possible limited impact of self-help programs [46].

To understand and possibly intervene in the process of non-adherence, our third objective was to study patients’ perceived barriers and facilitators to adhere to HM. Barriers to adhere to HM are comparable to results of other studies on prophylactic education and exercise programs, either home-based and/or institution-based, targeting patients who are about to undergo (C)RT for HNC [32,34]. Physical barriers to perform self-help exercises as identified in our study are in accordance with the findings of Kotz [34], who reported that 69% of HNC patients were unable to perform the swallowing exercises throughout the entire course of (C)RT because of oral pain, throat discomfort, and overall fatigue. Van der Molen [32] reported that 37% of HNC patients stopped training because of pain in the mouth, nausea, and fatigue. Additionally, Lagorio and Carnaby-Mann [47] reported that adherence of the preventative swallowing exercises was significantly associated with presence of depression and fatigue. Overall, it is important to realize that HNC patients often struggle to cope with the challenges of treatment while attempting to manage the other aspects of their lives such as work responsibilities, family issues, and social relationships [48]. HNC patients in our study reported time constraints, time consuming treatment protocols, being a caregiver, and distraction of daily routine as barriers to adhere to the HM exercises. These reports are consistent with previous qualitative studies [49,50]. During coaching sessions, exercise acceptance and adherence may be improved by paying attention to psychosocial factors [42,47], for example by providing e-counseling [51-53]. These counseling interventions deserve attention and may be uniquely beneficial as they spare patients the cost and burden of traveling to a hospital for psychosocial care [54]. However, the effects of such (psychosocial) interventions are yet unclear [55]. Future research is needed to assess the impact of (combined) coaching strategies (face-to-face, email, and/or telephone contact).

In the present study, several adherence facilitators related to the multimodal design of HM were identified, such as simple and easy-to-follow exercises, online or DVD demonstrations, the face-to-face introduction of HM, and the weekly coaching sessions. Efforts to enhance exercise adherence in HNC patients should focus on optimizing enjoyment while managing symptoms, providing education in overcoming treatment-related barriers, helpful types of support, self-monitoring, reminders, and telephone follow-up [56-58]. Other researchers suggest the use of a hook (a message or program design to build curiosity) to engage participants who are starting the intervention [49].

Results of earlier studies demonstrated the importance of the introduction session of a self-help program, to be able to achieve a successful dissemination. For health-related interventions, it is deemed crucial that the introduction is provided by a care professional who is a credible source for patients and who is committed to the program [59]. HM has the advantage that health care professionals have been involved in the development of the intervention, which increases commitment to the intervention and hereby the chance of optimal dissemination [60].

### Limitations

The outcome of our feasibility study provided support that a guided self-help exercise program during (C)RT is feasible. However, some limitations should be mentioned. The results were based on a relatively small sample size from a single center setting, which may have hampered the generalizability. Furthermore, no information was obtained from patients who refused to participate. Information from a non-participating group of patients would give a more balanced view of the perceived barriers to the HM program and to the feasibility of the program.

Another limitation was that exercise adherence and performance levels were reported on the basis of patients’ self-reported data (diaries). Use of paper diaries to capture patient experiences are favored due to familiarity, ease of use, low cost, and allowing locus-of-control by the patient. However, intentionally or not, many individuals may have difficulty keeping faithful records. Furthermore, the data may have been influenced by social desirability effects [61,62] and may not truly reflect the user’s experience and dose. Ideally, self-reports should be supplemented using alternative objective sources of data, such as usage statistics (number of log-ins, frequency, duration, activities completed, time spent online, pages opened), combined with qualitative measures, such as semistructured telephone interviews [63,64].

Though strengths of the present study include high uptake, the position of both the researcher and participant need to be considered. As typical of evaluative research, the interaction between an evaluator and participant may have produced an understanding that portrayed the feasibility in an excessively positive light. Hence, future research will focus on consistency of the barriers and facilitators perceived by participants with findings from quantitative analysis of adherence and the impact of different HM formats [65].

Finally, we explored barriers and facilitators to adherence. The qualitative nature of these data in this study did not enable us to identify the barriers and facilitators that would make the largest contribution to the adherence with and compliance to the self-help program. Further quantitative research is therefore needed.

### Conclusions

This feasibility study demonstrated that a multimodal guided self-help exercise program HM is feasible for HNC patients undergoing (C)RT. Feasibility of the exercise program in HNC patients is supported by high uptake (83%) and a reasonable adherence (64%). Several barriers (decreased physical condition, treatment-related barriers) and facilitators (increased physical condition, feeling motivated) were identified providing directions for future studies. Because HM is feasible, a study will be carried out investigating the (cost-)effectiveness of self-help exercises among HNC patients to prevent speech, swallowing, and shoulder problems after treatment.

## Multimedia Appendix 1

Overview of Head Matters: exercise categories and formats.

## Multimedia Appendix 2

Patient characteristics.

## Multimedia Appendix 3

Exercise performance levels.

## Multimedia Appendix 4

Patients' perceived barriers and facilitators to perform Head Matters.

## Abbreviations

(C)RT: (chemo)radiation
CRT: chemoradiation
HM: Head Matters
HNC: head and neck cancer
RT: radiotherapy
ST: speech and swallowing therapist
TNM: classification of Tumor size, lymph Nodes, and Metastasis
UICC: Union for International Cancer Control
