# Supplementary material for: Multimodal Guided Self-Help Exercise Program to Prevent Speech, Swallowing, and Shoulder Problems Among Head and Neck Cancer Patients: A Feasibility Study
Source: J Med Internet Res. 2014 Mar 6;16(3):e74. doi: 10.2196/jmir.2990 (PMC3961811; doi:10.2196/jmir.2990)
Supplement: Supplementary file 1 [file jmir_v16i3e74_app1.pdf]

Overview of Head Matters: exercise categories and formats

Exercise categories:

I) Exercises to maintain mobility of the head, neck and shoulders
- moving shoulders up and down, circling shoulders forward and backward
- bending head forward (‘Chin tuck’)
- looking up (‘Head back’)
- looking over left / right shoulder (‘Head turn’)
- touching ear to shoulder (‘Head tilt’)

II) Exercises and strategies to optimize, and to maintain swallowing function
- swallowing with strength (‘Effortful swallow’)
- pulling back of the tongue to the back of the mouth and hold (‘Tongue retraction’)
- swallowing two or three times per bite or sip
- taking sips of water regularly
- sit up straight during mealtimes, and minimize distractions during meals

III) Exercises to optimize vocal health, and to maintain vocal function
- humming with gradually increased volume, and with exagerated jaw movement
- slide up the pitch scale as high as possible; hold the high note for several seconds (‘Falsetto exercise’)

IV) Exercises to optimize speech function, and functional communication
- articulate each syllable
- stretching the tongue out straight
- pushing tongue against left / right cheek
- licking lips with the tip of the tongue
- maintaining eye contact during conversation

Formats:

(a) online [40] with general information about HNC, with written instructions, and photo and video demonstrations

(b) a 28-paged booklet with DVD, with general information about HNC, with written instructions, and pictorial and video demonstrations

(c) a two-paged A4 leaflet with written instructions
