# Supplementary material for: Multimodal Guided Self-Help Exercise Program to Prevent Speech, Swallowing, and Shoulder Problems Among Head and Neck Cancer Patients: A Feasibility Study
Source: J Med Internet Res. 2014 Mar 6;16(3):e74. doi: 10.2196/jmir.2990 (PMC3961811; doi:10.2196/jmir.2990)
Supplement: Supplementary file 2 [file jmir_v16i3e74_app2.pdf]

Patient characteristics

Gender	N (%)
Male	25 (76)
Female	8 (24)
Mean age in years	60 (10.62, 21-77) (SD, range)
Tumour site	
Oral cavity	8 (24)
Oropharynx	15 (46)
Hypopharynx	3 (9)
Larynx	7 (21)
Tumour classification (stage)	
I	5 (15)
II	2 (6)
III	8 (24)
IV	18 (55)
Treatment	
Radiotherapy	11 (33)
Chemoradiation	11 (33)
Postoperative (chemo-)radiation	11 (33)
