# Supplementary material for: Multimodal Guided Self-Help Exercise Program to Prevent Speech, Swallowing, and Shoulder Problems Among Head and Neck Cancer Patients: A Feasibility Study
Source: J Med Internet Res. 2014 Mar 6;16(3):e74. doi: 10.2196/jmir.2990 (PMC3961811; doi:10.2196/jmir.2990)
Supplement: Supplementary file 3 [file jmir_v16i3e74_app3.pdf]

Exercise performance levels

Performance level	Patient number[a]	Week number[b] 1	2	3	4	5	6	Total number of exercises performed
Low (4-167)	PT 10 ONLINE	0	0	0	4	0	0	4
	PT 4 ONLINE	0	0	8	0	0	0	8
	PT 17 BOOKLET	0	12	0	0	0	0	12
	PT 8 ONLINE	0	8	4	12	8	0	32
	PT 18 BOOKLET	0	0	11	11	9	5	36
	PT 7 ONLINE	0	0	0	27	21	3	51
	PT 33 LEAFLET	34	16	8	8	0	0	66
	PT 15 BOOKLET	34	7	10	8	7	7	73
	PT 12 BOOKLET	12	22	24	16	0	0	74
	PT 24 LEAFLET	20	28	28	0	0	0	76
	PT 32 LEAFLET	9	16	31	29	24	27	136
	PT 22 BOOKLET	16	27	29	25	26	30	153
	PT 29 LEAFLET	12	36	36	36	36	0	156
	PT 5 ONLINE	20	12	32	50	28	25	167
Moderate (196-332)	PT 20 BOOKLET	29	39	36	32	28	32	196
	PT 16 BOOKLET	48	70	28	28	28	12	214
	PT 13 BOOKLET	40	47	28	48	36	28	227
	PT 30 LEAFLET	48	48	51	40	34	10	231
	PT 26 LEAFLET	76	60	36	36	36	12	256
	PT 23 LEAFLET	36	84	80	25	21	35	281
	PT 11 ONLINE	0	28	36	84	84	81	313
	PT 9 ONLINE	0	24	50	76	80	84	314
	PT 6 ONLINE	39	55	65	56	59	46	320
	PT 19 BOOKLET	48	84	84	36	24	56	332
High (372-495)	PT 14 BOOKLET	48	84	42	42	84	72	372
	PT 28 LEAFLET	40	71	74	76	76	56	393
	PT 31 LEAFLET	12	84	84	84	84	48	396
	PT 27 LEAFLET	18	84	84	84	84	48	402
	PT 3 ONLINE	60	69	69	78	76	52	404
	PT 25 LEAFLET	48	84	84	80	69	47	412
	PT 1 ONLINE	49	80	84	84	66	60	423
	PT 2 ONLINE	72	84	84	84	84	84	492
	PT 21 BOOKLET	84	84	84	84	84	75	495

a HM via leaflet (n=11), booklet (n=11), or online (n=11)

b = non-active (no exercises per week)
= highly active (maximum number exercises per week)
