# Supplementary material for: Multimodal Guided Self-Help Exercise Program to Prevent Speech, Swallowing, and Shoulder Problems Among Head and Neck Cancer Patients: A Feasibility Study
Source: J Med Internet Res. 2014 Mar 6;16(3):e74. doi: 10.2196/jmir.2990 (PMC3961811; doi:10.2196/jmir.2990)
Supplement: Supplementary file 4 [file jmir_v16i3e74_app4.pdf]

Patients' perceived barriers and facilitators to perform Head Matters

	BARRIERS	FACILITATORS
Physical condition	Decreased condition of the head and neck<br>- oral complications<br>- throat discomfort<br>- stiffness in neck and shoulders<br><br>Decreased general condition<br>- pain<br>- nausea<br>- weight loss<br>- fatigue	Increased condition of the head and neck<br>- increased appetite<br>- better vocal function<br>- decrease of tumour (size)<br><br>Increased general condition<br>- having regained energy
Treatment related factors	Treatment related barriers<br>- hospitalization<br>- time consuming treatment protocols<br>- great time loss travelling to hospital	
Psychological functioning	Emotional problems<br>- anxiety<br>- worrying<br>- panic attacks<br>- finding it difficult to focus	General sense of psychological well being<br>- feeling good<br>- being good-humoured
Motivational issues	Lack of motivation<br>- experiencing (no) complaints<br>- lack of confidence that exercising will help<br>- no preference for a self-help program<br>- distraction of daily routine<br>- perceived information overload	Motivational facilitators<br>- simple and easy-to follow exercises<br>- experiencing (direct) benefits of exercising<br>- adapt exercises to one's own ability<br>- enjoying the exercise program with weekly coaching sessions and a face-to-face introduction
Social issues	Social barriers at home<br>- time constraints and inability to focus because of being a caregiver<br><br>Social barriers at work<br>- time constraints and inability to focus because of high workload	Social facilitators at home<br>- partner and patient are performing exercises together<br>- partner encourages patient to perform the exercises<br>- partner and family motivate by reporting improved speech function<br><br>Social facilitators at work<br>- performing exercises while being at work<br>- off duty: time to perform the exercises
Technical issues	Technical problems<br>- not able to see the videos on the computer<br>- exercises on DVD take too much time<br>- failure to remember login password or website address	Technical benefits<br>- online or DVD exercise demonstrations make it easy to perform the exercises as instructed
